# The genetic basis of natural variation for iron homeostasis in the maize IBM population

**DOI:** 10.1186/1471-2229-14-12

**Published:** 2014-01-09

**Authors:** Andreas Benke, Claude Urbany, Johanna Marsian, Rongli Shi, Nicolaus von Wirén, Benjamin Stich

**Affiliations:** 1 Max Planck Institute for Plant Breeding Research, Carl-von-Linné Weg 10, 50829 Köln, Germany; 2 Leibniz Institute of Plant Genetics and Crop Plant Research, Corrensstraße 3, 06466 Gatersleben, Germany

## Abstract

**Background:**

Iron (Fe) deficiency symptoms in maize (*Zea mays* subsp. *mays*) express as leaf chlorosis, growth retardation, as well as yield reduction and are typically observed when plants grow in calcareous soils at alkaline pH. To improve our understanding of genotypical variability in the tolerance to Fe deficiency-induced chlorosis, the objectives of this study were to (i) determine the natural genetic variation of traits related to Fe homeostasis in the maize intermated B73 × Mo17 (IBM) population, (ii) to identify quantitative trait loci (QTLs) for these traits, and (iii) to analyze expression levels of genes known to be involved in Fe homeostasis as well as of candidate genes obtained from the QTL analysis.

**Results:**

In hydroponically-grown maize, a total of 47 and 39 QTLs were detected for the traits recorded under limited and adequate supply of Fe, respectively.

**Conclusions:**

From the QTL results, we were able to identify new putative candidate genes involved in Fe homeostasis under a deficient or adequate Fe nutritional status, like Ferredoxin class gene, putative ferredoxin *PETF*, metal tolerance protein *MTP4*, and *MTP8*. Furthermore, our expression analysis of candidate genes suggested the importance of *trans*-acting regulation for 2’-deoxymugineic acid synthase 1 (*DMAS1*), nicotianamine synthase (*NAS3, NAS1*), formate dehydrogenase 1 (*FDH1*), methylthioribose-1-phosphate isomerase (*IDI2*), aspartate/tyrosine/aromatic aminotransferase (*IDI4*), and methylthioribose kinase (*MTK*).

## Background

Iron (Fe) deficiency in maize (*Zea mays* subsp. *mays*) mostly occurs during growth on calcareous or alkaline soils, where Fe becomes sparingly soluble due to its precipitation in form of hydroxides, oxides, or phosphates [1]. Approximately 30% of the world’s arable soils are of high pH and include preferential maize cultivation areas like the river valley of Nebraska with about 0.4 million hectares (ha) [2] and the arid and semi-arid regions of the Great Plains [3-5]. Therefore, yield reduction in Fe-deficient maize is of agronomic importance [6].

Graminaceous plant species like maize acquire Fe by so-called strategy II mechanisms, which include the release of phytosiderophores, acting as high-affinity hexadentate chelators for ferric Fe, and an elevated expression of transport systems for Fe(III)-phytosiderophores at the root plasma membrane [7]. Comparative studies among different grass species have suggested that in particular the release of phytosiderophores is limiting most an efficient acquisition of Fe from the soil [8]. Relative to other graminaceous plant species, however, maize is generally considered as a weak strategy II plant, i.e. releasing approximately fivefold lower amounts of phytosiderophores than for instance barley [9]. This may explain at least in part the high susceptibility of maize to Fe deficiency-induced chlorosis. Although genotypical variation for chlorosis tolerance and the rate of phytosiderophore release have already been reported [10], attempts to characterize the intraspecific variation in chlorosis tolerance or other Fe efficiency traits across larger populations of maize genotypes have not yet been reported.

Previous studies on other graminaceous plant species have characterized various essential mechanisms and genes involved in Fe efficiency, i.e. the ability of plants to produce less chlorotic leaves, higher biomass, or grain yield under Fe-deficient growth conditions. In graminaceous species Fe deficiency upregulates the transcription factors IDEF1 and IRO2, which leads to an increase of phytosiderophore biosynthesis [11]. The first step in phytosiderohore biosynthesis is the conjugation of three *S*-adenosyl-methionine (SAM) residues by the enzyme nicotianamine synthase (NAS) to nicotianamine (NA) [12]. The linked methionine salvage pathway restores methionine levels and includes the genes methylthioadenosine/S-adenosyl homocysteine nucleosidase (*MTN*), methylthioribose kinase (*MTK*), methylthioribose-1-phosphate isomerase (*IDI2*), dehydratase-enolase-phosphatase (*DEP*), aspartate/tyrosine/aromatic aminotransferase (*IDI4*), and Formate dehydrogenase 1 *FDH* restores the Methionine for SAM synthesis [13]. NA is then subject to subsequent amino transfer by nicotianamine aminotransferase (NAAT) [14] and a reduction step by deoxymugineic acid synthase (DMAS) to yield deoxy-mugineic acid (DMA), which is the only phytosiderophore species being released by maize plants [15]. Subsequently, DMA is released by the transporter of mugineic acid 1 (TOM1) which is localized at the root plasma membrane [16]. Most of these genes being involved in phytosiderophore biosynthesis and release are subject to upregulation when the Fe nutritional status of the shoot is low [17]. Following metal chelation in the rhizosphere, the uptake of Fe(III)-phytosiderophores into root cells is mediated by membrane proteins of the yellow stripe 1/yellow stripe like (YS1/YSL) family that possess a particularly high affinity for phytosiderophore-chelated ferric Fe [18,19]. The fairly robust upregulation of *YS1* gene expression under Fe-deficiency goes along with an upregulation of ST-type sulfate transporters, most likely due to an enhanced sulphur demand for synthesis of the nicotianamine precursor SAM [20]. Inside root cells, ferric Fe may be reduced and exchange chelated to NA [21] and further transported radially for xylem loading and translocated to the shoot, where the majority of Fe is stored in ferritin (FER). In seeds and young seedlings vacuolar loading and unloading are critical for Fe efficiency too, since Fe loading of the vacuole by the vacuolar iron transporter VIT1 [22] and remobilization therefrom by natural resistance associated macrophage proteins (NRAMP3 and 4) are at least in Arabidopsis essential processes for early seedling development under Fe-limiting growth conditions [23]. While in Arabidopsis and other plants VIT1 is poorly responsive to the Fe nutritional status of the plant, NRAMP3 and NRAMP4 are upregulated under Fe deficiency [22,23].

Despite this comprehensive knowledge on the functional aspects of Fe acquisition and homeostasis, studies examining the natural variation of Fe efficiency traits in maize and the role of natural allelic variation in determining bottle-necks of Fe efficiency in maize have remained poor. However, such information will be instrumental for the selection and development of chlorosis-tolerant maize cultivars by classical plant breeding methods. Furthermore, when such analyses are linked to molecular marker information, they have the potential to identify new genes mechanistically involved in the trait of interest, that have not been identified using classical functional genetics. This is due to the fact that in contrast to mutant screens, which consider one gene in one genetic background [24], analyses on the natural variation of traits allow discovering multiple gene actions in complex genetic backgrounds [25].

One first step to reach this goal is quantitative trait loci (QTL) mapping, which provides information on the chromosomal locations contributing to the quantitative variation of complex traits [26,27]. Besides high resolution mapping of such QTLs, their combination with expression studies of positional candidate genes have the potential to improve our understanding of the QTL of interest.

The objectives of this study were to (i) determine the natural genetic variation of traits related to Fe homeostasis in the maize intermated B73 × Mo17 (IBM) population when these plants were grown under adequate or limiting Fe supply, (ii) identify QTLs for these traits, (iii) analyze Fe-dependent expression levels of genes known to be involved in Fe homeostasis as well as positional candidate genes from QTL analysis.

## Results

### Heritability

The heritability represents the genotypic contribution to the phenotypic variation. The variance analysis of the phenotypic data evaluated for each of the 13 traits using 85 intermated recombinant inbred lines (IRILs) provided the genetic and error variance values for the heritability calculation. The broad sense heritabilities for the traits evaluated under Fe deficiency ranged from 0.35 (shoot length (SL)) to 0.80 (SPAD value at leaf 3 (SP3)) (Table 1). A similar trend was observed for the broad sense heritabilities for the Fe-sufficient growth regime, which was lowest for SL (0.28) and highest for SP5 (0.80).

**Table 1:** **Traits recorded in the current study for two Fe conditions (Fe-deficient and Fe-sufficient), where** ***H***^***2***^** is the broad sense heritability on an entry means basis**

			***H***^**2**^	
**Trait**	**Abbreviation**	**Unit**	**Fe-deficient**	**Fe-sufficient**
SPAD value at leaf 3	SP3		0.80	0.67
SPAD value at leaf 4	SP4		0.77	0.70
SPAD value at leaf 5	SP5		0.80	0.80
SPAD value at leaf 6	SP6		0.75	0.64
Root length	RL	cm	0.51	0.42
Root weight	RW	g	0.66	0.50
Shoot length	SL	cm	0.35	0.28
Shoot dry weight	SDW	g	0.58	0.38
Shoot water content	H_2_O	%	0.65	0.65
Ratio between shoot dry weight and shoot length	SDW/SL	g cm^-1^	0.53	0.41
Branching at the terminal 5 cm of root	BTR		0.64	^1^
Lateral root formation	LAT		0.55	0.58
Leaf necrosis	NEC		0.44	0.59

For details, see materials and methods.

^1^No variation observed.

### Trait variation in the IBM population

**Figure 1:**
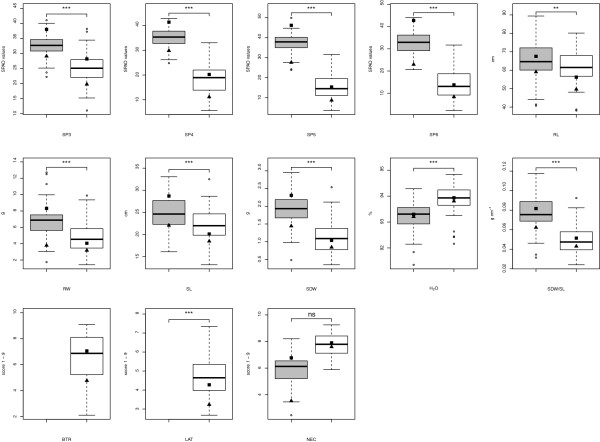
**Boxplot of the adjusted entry means of all traits for the 85 maize intermated recombinant inbred lines of the maize IBM population evaluated at Fe-deficient (white) and Fe-sufficient (grey) regimes.** The adjusted entry means of the parental inbreds B73 and Mo17 are represented by a square and a triangle, respectively. The line in the box represents the median of the trait. T-test application to examine the difference for a trait between both Fe conditions. *, **, ***: *P* = 0.05, 0.01, and 0.001, respectively; ns, not significant.

The adjusted entry means (AEM) for the traits SP3 to SP6, root length (RL), root weight (RW), SL, shoot dry weight (SDW), SDW/SL, and lateral root formation (LAT) was on average across all IRILs lower under Fe deficiency than under Fe sufficiency (Figure 1). The trait water (H_2_O) showed higher AEM values for the IRILs under Fe deficiency, whereas leaf necrosis (NEC) showed on average across all IRILs no difference between both Fe regimes. The parental inbred Mo17 showed for all traits in both Fe regimes a lower AEM compared to the second parental inbred B73. The progenies showed transgressive segregation for all traits.

**Figure 2:**
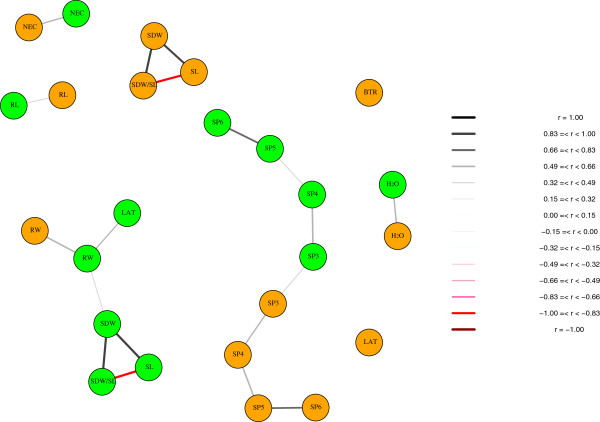
**Network representation of the partial correlations between all pairs of traits evaluated at Fe-deficient (orange) and Fe-sufficient (green) conditions for the 85 maize intermated recombinant inbred lines.** Thickness and color intensity of the lines is proportional to the strength and direction of the partial correlation.

The network analysis based on the partial correlation coefficients between all pairs of traits that focus on the correlation between the residuals revealed eight groups of traits (Figure 2). The highest positive and negative correlation was observed between SDW and SDW/SL (between 1.00 and 0.83) and between SDW/SL and SL (between -1.00 and -0.83), respectively, for both Fe regimes. Furthermore, the Pearson pairwise correlation coefficients between the SPAD value and the shoot Fe concentration increased from SP3 (0.41), SP4 (0.51), SP5 (0.61) to SP6 (0.62) under Fe deficiency. In contrast, the opposite trend was observed under Fe sufficiency where the correlation coefficients ranged from 0.08 (SP3), 0.01 (SP4), -0.28 (SP5) to -0.41 (SP6).

### QTL analysis

The QTL analyses for the traits recorded under Fe deficiency regime using the 85 IRILs and their corresponding AEMs for each trait revealed a total of 47 QTLs (Table 2). The highest number of QTLs was detected for SP3 (8) and the lowest for RL, SL, SDW/SL, and LAT (1). The proportion of phenotypic variance explained by the QTL was highest for SP5 QTL2 (34.0%). The maximum of the proportion of phenotypic variance explained in a simultaneous fit by all QTLs for one trait was 59.4% (SP3), where the minimum was 9.6% (SL). The additive effect of the QTLs revealed that at 15 QTLs the allele increasing the trait value was contributed by Mo17.

**Table 2:** **Summary of the quantitative trait loci (QTL) detected using the maize IBM population evaluated in a Fe-deficient nutrient solution, where Chr. is the chromosome, Pos. the position in centi Morgan on the genetic map, Add. the additive effect, %r**^**2**^** the percentage of the explained phenotypic variance, and genetic map interval in centi Morgan of the flanking markers with corresponding physical map interval including the number of genes in the corresponding QTL confidence interval according to the filtered gene set B73 RefGen_v2**

**Trait**	**QTL**	**Chr.**	**Pos. (cM)**	**Add.**	**%r**^**2**^	**Interval (cM)**			**Flanking markers**			**Physical map interval**			**Genes**
SP3	1	1	556.0	1.5	1.7	553.6	-	557.6	umc1748	-	bnlg1615	191,860,023	-	192,968,163	17
SP3	2	2	524.0	3.1	8.3	523.5	-	529.2	umc1604	-	bnlg1316	211,345,382	-	212,352,714	29
SP3	3	4	226.0	0.2	< 0.1	225.7	-	228.4	umc1963	-	umc1652	26,437,539	-	27,757,462	22
SP3	4	4	240.0	3.1	2.8	237.8	-	245.5	bnlg490	-	agrr301	31,323,581	-	40,458,411	186
SP3	5	7	252.0	4.1	13.7	249.1	-	252.4	umc1929	-	umc1787	105,804,341	-	110,057,749	42
SP3	6	8	464.0	4.1	15.0	460.8	-	464.0	bnlg1065	-	rz538a	165,689,209	-	166,244,750	20
SP3	7	9	206.0	-1.5	0.9	204.4	-	208.5	ufg71	-	mmp170b	25,825,748	-	26,709,137	19
SP3	8	9	222.0	4.2	7.2	220.7	-	223.9	psr160c	-	rz273c	26,822,048	-	49,039,937	292
				Total	59.4										
SP4	1	1	834.0	6.1	21.9	833.0	-	839.3	chrom7	-	glb1	256,342,909	-	257,540,930	26
SP4	2	4	278.0	4.1	5.8	277.8	-	279.9	psr152b	-	nnr1	46,450,572	-	65,900,096	202
SP4	3	4	300.0	0.2	< 0.1	299.9	-	300.2	bnlg1755	-	mmp45	118,324,214	-	135,333,950	173
SP4	4	8	194.0	-3.2	6.3	191.0	-	194.1	mmp120	-	mmp72	23,404,908	-	60,338,399	367
				Total	40.1										
SP5	1	1	690.0	-4.6	10.3	685.2	-	690.5	lim442	-	mmp189	214,921,545	-	219,064,269	68
SP5	2	1	838.0	8.4	34.0	833.0	-	839.3	chrom7	-	glb1	256,342,909	-	257,540,930	26
SP5	3	4	238.0	5.1	14.0	237.8	-	245.5	bnlg490	-	agrr301	31,323,581	-	40,458,411	186
				Total	49.4										
SP6	1	1	690.0	-3.8	4.9	685.2	-	690.5	lim442	-	mmp189	214,921,545	-	219,064,269	68
SP6	2	1	714.0	-2.3	1.7	711.5	-	714.4	umc1128	-	umc1147	224,265,940	-	224,970,667	18
SP6	3	1	830.0	7.7	27.7	825.8	-	833.0	rz403	-	chrom7	255,041,502	-	257,540,930	53
SP6	4	4	238.0	5.2	14.1	237.8	-	245.5	bnlg490	-	agrr301	31,323,581	-	40,458,411	186
				Total	44.2										
RL	1	3	452.0	-6.9	18.0	451.5	-	452.7	jpsb79	-	umc60	180,725,934	-	180,867,611	4
				Total	18.0										
RW	1	1	824.0	0.5	0.8	821.5	-	825.8	csu696	-	rz403	253,570,111	-	256,342,908	57
RW	2	1	846.0	0.8	1.9	839.3	-	847.3	glb1	-	csu222a	256,342,909	-	261,572,322	145
RW	3	5	74.0	1.3	12.6	73.3	-	74.4	mmp43	-	bnl7.21c	3,727,289	-	3,810,656	6
RW	4	5	412.0	1.2	11.0	410.8	-	413.6	umc1155	-	csu173	180,186,573	-	181,568,742	33
RW	5	7	148.0	0.7	3.7	132.0	-	148.5	asg34a	-	gta101a	14,027,268	-	14,698,304	10
RW	6	7	290.0	0.9	5.9	288.9	-	298.4	umc116a	-	umc1713	127,039,567	-	129,866,479	59
RW	7	8	194.0	-0.9	6.7	191.0	-	194.1	mmp120	-	mmp72	23,404,908	-	60,338,399	367
				Total	58.9										
SL	1	1	840.0	2.4	9.6	839.3	-	847.3	glb1	-	csu222a	256,342,909	-	261,572,322	145
				Total	9.6										
SDW	1	1	888.0	0.3	11.1	887.5	-	890.9	cdo122a	-	AY110019	263,205,925	-	270,965,223	186
SDW	2	4	238.0	0.3	11.9	237.8	-	245.5	bnlg490	-	agrr301	31,323,581	-	40,458,411	186
SDW	3	7	164.0	0.1	0.4	162.4	-	167.4	AY105589	-	npi600	17,029,068	-	21,464,802	82
SDW	4	7	174.0	-0.1	< 0.1	170.8	-	178.0	crt2	-	AY110473	24,318,258	-	50,154,299	312
SDW	5	7	184.0	0.3	1.4	183.7	-	184.4	uaz187	-	mmp26	50,078,806	-	50,149,169	3
				Total	34.6										
H_2_O	1	2	370.0	-0.3	8.0	369.3	-	373.5	umc1079	-	bnlg1036	152,207,394	-	163,566,033	150
H_2_O	2	4	272.0	-0.4	8.8	271.4	-	274.7	umc1964	-	AY110290	42,102,039	-	46,621,469	69
H_2_O	3	4	300.0	-0.1	0.9	299.9	-	300.2	bnlg1755	-	mmp45	118,324,214	-	135,333,950	173
H_2_O	4	9	138.0	0.3	0.8	131.1	-	139.0	omt2	-	mmp162	15,578,721	-	18,071,240	91
H_2_O	5	9	146.0	-0.7	1.7	142.6	-	147.5	bnlg244	-	bnlg1401	18,040,440	-	18,071,240	0
H_2_O	6	9	150.0	0.6	2.0	147.5	-	153.0	bnlg1401	-	mmp77	18,561,278	-	18,607,113	0
H_2_O	7	9	164.0	0.1	0.6	162.5	-	170.4	mmp30	-	umc1698	16,660,671	-	20,791,656	146
				Total	35.9										
SDW/SL	1	8	466.0	< 0.1	14.4	464.0	-	466.5	rz538a	-	umc1607	165,636,122	-	166,188,782	20
				Total	14.4										
BTR	1	1	672.0	-1.1	7.3	670.2	-	685.2	umc23a	-	lim442	212,899,665	-	219,195,665	110
BTR	2	1	836.0	1.9	20.9	833.0	-	839.3	chrom7	-	glb1	256,342,909	-	257,540,930	26
BTR	3	8	156.0	1.0	6.4	153.3	-	156.6	umc1974	-	psr598	16,722,932	-	18,272,098	35
				Total	36.7										
LAT	1	8	188.0	-0.8	11.8	179.5	-	191.0	umc1530	-	mmp120	22,245,756	-	25,351,500	59
				Total	11.8										
NEC	1	1	208.0	-0.8	19.0	205.0	-	208.5	lim122	-	umc1073	27,398,858	-	32,868,895	125
NEC	2	2	90.0	0.6	12.6	87.8	-	90.3	BE640649	-	npi421a	6,474,435	-	6,534,813	1
				Total	24.0										

Total is the percentage of the phenotypic variation explained by all QTL for a trait in a simultaneos fit.

Under Fe sufficiency, 39 QTLs were detected (Table 3). In dependence of the individual trait, the number of QTLs ranged from 10 (SP5) to 1 (SP4 and SL). The proportion of phenotypic variance explained by the QTL showed for QTL2 of SDW the highest (21.8%) value. The proportion of phenotypic variance explained in a simultaneous fit by all QTLs was maximal for SP5 (65.5%) and minimal for SL (9.8%). The additive effect of the QTLs indicated for 12 QTLs that the trait increasing allele was contributed by Mo17.

**Table 3:** **Summary of the quantitative trait loci (QTL) detected using the maize IBM population evaluated in a Fe-sufficient nutrient solution, where Chr. is the chromosome, Pos. the position in centi Morgan on the genetic map, Add. the additive effect, %r**^**2**^** the percentage of the explained phenotypic variance, and genetic map interval in centi Morgan of the flanking markers with corresponding physical map interval including the number of genes in the corresponding QTL confidence interval according to the filtered gene set B73 RefGen_v2**

**Trait**	**QTL**	**Chr.**	**Pos. (cM)**	**Add.**	**%r**^**2**^	**Interval (cM)**			**Flanking markers**			**Physical map interval**			**Genes**
SP3	1	1	852.0	3.2	14.8	847.3	-	864.6	csu222a	-	umc197a	257,951,442	-	262,818,614	141
SP3	2	3	508.0	1.9	0.6	507.2	-	511.1	AY111125	-	php15033	190,454,440	-	193,733,670	72
SP3	3	3	516.0	1.2	0.2	512.7	-	517.0	AI770795	-	pco067132	193,638,925	-	193,733,670	0
SP3	4	4	272.0	2.2	8.2	271.4	-	274.7	umc1964	-	AY110290	42,102,039	-	46,621,469	69
				Total	42.9										
SP4	1	1	840.0	3.8	21.5	839.3	-	847.3	glb1	-	csu222a	256,342,909	-	261,572,322	145
				Total	21.5										
SP5	1	1	840.0	2.2	2.2	839.3	-	847.3	glb1	-	csu222a	256,342,909	-	261,572,322	145
SP5	2	1	864.0	2.2	2.4	847.3	-	864.6	csu222a	-	umc197a	257,951,442	-	262,818,614	141
SP5	3	3	518.0	3.2	10.2	517.0	-	520.7	asg7b	-	bnl6.16a	190,889,172	-	196,152,996	138
SP5	4	4	274.0	2.1	4.3	271.4	-	274.7	umc1964	-	AY110290	42,102,039	-	46,621,469	69
SP5	5	7	266.0	3.2	5.9	265.3	-	280.5	mmp21	-	ufg54	121,188,675	-	129,322,867	172
SP5	6	7	286.0	0.8	0.4	285.4	-	288.9	cdo412b	-	umc116a	128,981,461	-	130,295,570	28
SP5	7	9	254.0	2.7	8.4	253.7	-	254.0	AW257883	-	umc1743	100,609,365	-	100,724,531	3
SP5	8	10	258.0	1.9	1.4	256.8	-	259.4	AY109920	-	AY109876	107,077,362	-	108,377,749	17
SP5	9	10	270.0	0.6	0.1	269.6	-	271.3	mmp121	-	AY110365	113,828,273	-	114,695,818	18
SP5	10	10	288.0	0.5	0.1	287.9	-	290.9	umc1330	-	umc1697	122,801,569	-	122,924,003	3
				Total	65.5										
SP6	1	1	698.0	-2.5	1.7	693.6	-	699.9	mmp173	-	php20661	222,399,299	-	222,711,359	10
SP6	2	1	706.0	-0.4	0.1	703.5	-	706.4	bcd207a	-	AY110356	223,974,395	-	224,078,859	3
SP6	3	1	862.0	2.3	1.9	847.3	-	864.6	csu222a	-	umc197a	257,951,442	-	262,818,614	141
SP6	4	1	884.0	1.3	0.6	882.7	-	886.1	tb1	-	umc1431	266,933,205	-	267,050,083	4
SP6	5	4	210.0	0.9	0.4	189.1	-	211.4	mmp111	-	npi386a	17,981,907	-	25,233,582	114
SP6	6	4	226.0	2.9	5.1	225.7	-	228.4	umc1963	-	umc1652	26,437,539	-	27,757,462	22
SP6	7	4	414.0	2.5	6.1	411.3	-	414.2	umc2038	-	umc19	172,796,153	-	173,318,462	19
SP6	8	8	622.0	3.0	8.9	621.6	-	626.7	umc1638	-	umc1916	174,236,946	-	175,350,404	34
SP6	9	9	224.0	2.2	4.6	223.9	-	226.3	rz273c	-	umc81	27,011,615	-	27,062,858	2
				Total	62.3										
RW	1	5	72.0	1.2	2.0	71.9	-	73.3	umc1523	-	mmp43	3,320,773	-	3,727,971	30
RW	2	5	80.0	0.3	0.1	74.4	-	83.7	bnl7.21c	-	jpsb239a	3,320,773	-	4,383,850	70
RW	3	7	290.0	1.5	13.6	288.9	-	298.4	umc116a	-	umc1713	127,039,567	-	129,866,479	59
				Total	25.4										
SL	1	1	1016.0	-2.4	9.8	1014.9	-	1031.8	igl1	-	umc2242	287,881,695	-	290,146,916	70
				Total	9.8										
SDW	1	1	864.0	0.3	10.7	847.3	-	864.6	csu222a	-	umc197a	257,951,442	-	262,818,614	141
SDW	2	7	534.0	-0.4	21.8	533.7	-	536.7	npi380	-	npi433	168,366,640	-	169,262,520	36
				Total	31.4										
H_2_O	1	2	154.0	-0.3	6.8	153.1	-	154.6	umc1262a	-	umc1261a	13,716,641	-	14,406,715	20
H_2_O	2	4	402.0	-0.3	5.6	397.4	-	408.7	umc66a	-	umc104a	162,903,780	-	172,858,882	273
H_2_O	3	6	69.3	-0.2	2.9	70.2	-	70.3	umc1606	-	cdo1173c	9,410,989	-	21,903,419	132
H_2_O	4	7	258.0	-0.4	9.3	252.9	-	261.5	umc2092	-	umc5b	109,977,320	-	122,636,428	186
				Total	37.0										
NEC	1	3	418.0	-0.7	11.7	416.1	-	423.6	asg39	-	BE639846	175,604,963	-	176,553,867	33
NEC	2	7	532.0	-0.6	4.8	518.9	-	532.0	umc1412	-	umc245	167,566,759	-	169,345,697	73
NEC	3	7	542.0	-0.1	0.2	540.8	-	543.4	mmp67	-	mmp25	169,262,253	-	170,497,141	42
NEC	4	7	588.0	- < 0.1	< 0.1	586.6	-	598.9	cdo938d	-	umc1406	170,246,381	-	170,998,616	39
NEC	5	7	602.0	0.6	2.1	600.4	-	602.9	umc2334	-	ufg39	170,246,381	-	170,998,616	39
				Total	29.5										

Total is the percentage of the phenotypic variation explained by all QTL for a trait in a simultaneos fit.

The largest QTL confidence interval was detected for QTL5 of RW (16.5 cM) and the lowest for QTL3 of SP4 and H_2_O (0.3 cM) under Fe deficiency. For Fe sufficiency, the size of the confidence intervals ranged from 22.3 cM for QTL5 of SP6 to 0.1 cM for QTL3 of H_2_O. The number of genes detected under Fe deficiency and Fe sufficiency within these confidence intervals using the physical map information ranged from 367 (SP4 QTL4 and RW QTL7) to 0 (H_2_O QTL5 and QTL6) and between 273 (QTL2 of H_2_O) and 0 (QTL3 of SP3), respectively.

### QTL confidence interval projection

**Figure 3:**
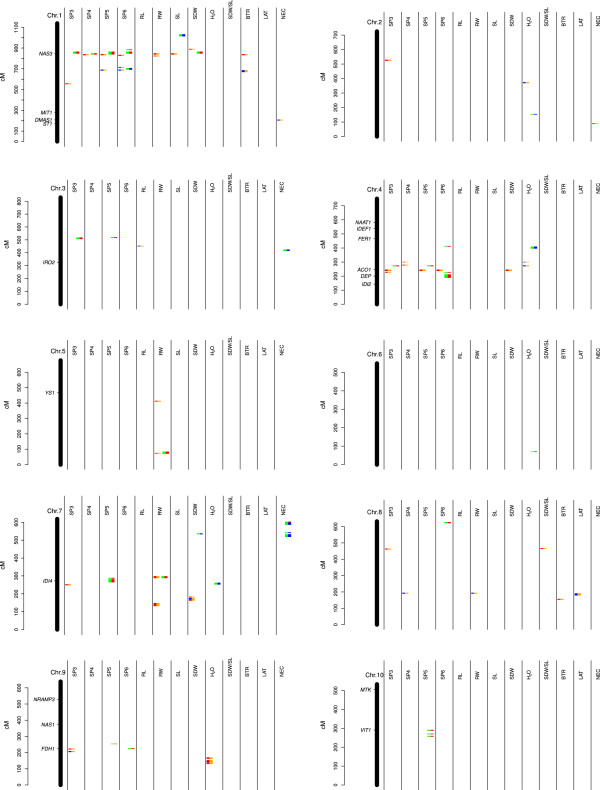
**Projection of 18 genes involved in iron homeostasis on the IBM2 genetic map.** The genetic position of confidence intervals of the quantitative trait loci detected for the 13 traits of our study at Fe-deficient and Fe-sufficient regimes are represented by orange and green bars, respectively. The trait value increasing alleles determined according to QTL analyses are indicated as blue and red bars for Mo17 and B73, respectively.

The presentation of the QTL confidence intervals on the genetic map revealed for regions on chromosome 1, 4, 7, and 8 a clustering of QTLs for multiple traits (Figure 3). We observed that the genes involved in Fe homeostasis *NAS3*, *MTN*, Aconitase 1 *ACO1*, *DEP*, *IDI4*, *FDH1*, and *VIT1* mapped to QTL confidence intervals.

### Expression analysis

**Figure 4:**
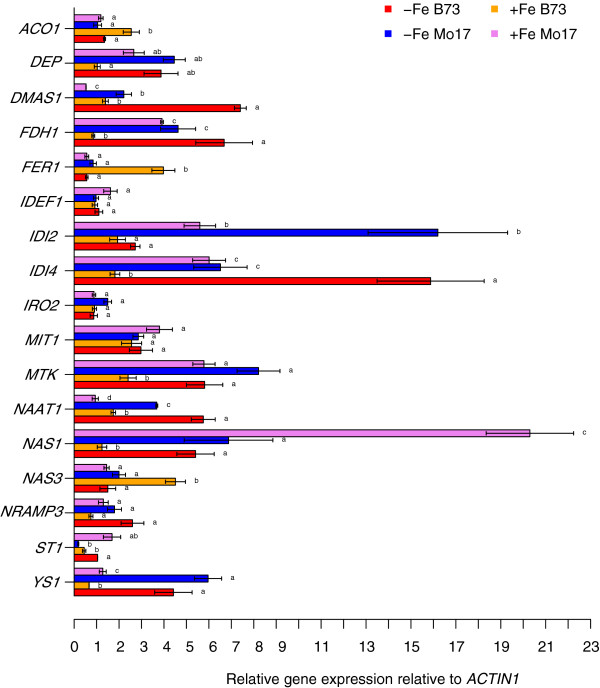
**Quantitative transcript levels of 17 genes important for Fe homeostasis under Fe-deficient (-Fe) and Fe-sufficient (+Fe) regimes ± standard error relative to the transcript level of *ACTIN1*.** Mean values of four technical replications of the parental inbred lines B73 and Mo17 marked with different letters for each gene are significantly (*α* = 0.05) different expressed.

The expression levels of genes relative to *ACTIN1* ranged between 0.19 (Mo17 *ST1*) and 16.20 (Mo17 *IDI2*) under Fe deficiency and between 0.45 (B73 *ST1*) and 20.30 (Mo17 *NAS1*) under Fe sufficiency (Figure 4). No significant differences (*α* = 0.05) between both Fe regimes were observed for *IDEF1, IRO2, mitochondrial iron transporter 1 MIT1* and *NRAMP3*, whereas expression of the remaining genes was significantly different (*α* = 0.05). The strongest expression differences between B73 and Mo17 under Fe deficiency were detected for *DMAS1, IDI2, IDI4* and *MTK*. Furthermore, under Fe sufficiency striking differences were observed for *FDH1, FER1, IDI2, IDI4, MTK, NAS1*, and *NAS3*.

## Discussion

Maize is often grown on soils with low Fe availability although it is highly sensitive to Fe deficiency-induced chlorosis. Therefore, understanding the genetic architecture of Fe-efficiency in maize is instrumental for the selection and development of chlorosis-tolerant maize cultivars by classical plant breeding methods. Furthermore, when such analyses are linked to molecular marker information they have the potential to identify new genes mechanistically involved in the trait of interest. Therefore, we took a two-step approach to explore the intraspecific variation in Fe-responsive traits in a segregating population of maize. First, traits related to Fe homeostasis were determined that promise to be relevant for Fe-efficiency. In a second step, these traits were used for a quantitative genetic approach and the subsequent determination of new candidate genes involved in Fe homeostasis.

### Genotypic variation of traits related to Fe homeostasis

The means of all traits, except NEC, showed a highly significant (*α* = 0.01) difference between the two examined Fe regimes (Figure 1). This finding is in accordance with results of [28] who observed a considerable reduction in biomass and chlorophyll concentration under Fe-deficient growth conditions. Our observation illustrates the significant influence of Fe supply on the extent of phenotypical changes in the IBM population.

The broad sense heritabilities observed for the traits under consideration were moderate to high at both Fe regimes (Table 1). This is in accordance with the results of [29] who detected high heritabilities for Fe concentration in maize kernels of the IBM population. This observation indicates that the data of our study provide a reliable basis for detecting QTLs for morphological and physiological traits contributing to superior plant performance under different Fe regimes [30].

To verify whether the SPAD value could be used as indirect measure for the Fe nutritional status, we correlated the leaf chlorophyll index (SPAD value) with measured Fe concentrations. Plants grown under adequate Fe supply showed no correlation between leaf greenness measured by SPAD and the Fe concentration. This is not surprising considering the facts that i) Fe partially precipitates in the apoplast contributing to the so-called chlorosis paradox [31], and ii) the chlorophyll concentration decreases under Fe overload in the chloroplast [32]. When Fe provision to shoots is sufficient to saturate chlorophyll biosynthesis, excess Fe will be stored in ferritin [33] to prevent chlorophyll degradation.

In contrast to Fe-sufficient growth conditions, a correlation coefficient of 0.41 to 0.62 was observed for the correlation between Fe concentrations in leaves and the SPAD values obtained from Fe-deficient plants. This finding was in accordance with results of [28] who observed a tight relationship between the chlorophyll concentration and the extractable Fe in maize leaves under Fe-limiting conditions. This relationship indicates that the SPAD values measured in our study were appropriate measures of the Fe nutritional status under Fe-deficient conditions.

With respect to the SPAD measurements, the 3^*rd*^ leaf of Fe-deficient plants showed higher trait values in comparison to the younger leaves 4 to 6 (Figure 1). Furthermore, the 3^*rd*^ leaf exhibited the smallest differences between the Fe-deficient and Fe-sufficient regimes. Under Fe-sufficiency, no obvious difference between the means of SP3 to SP6 was observed. Only for leaf no. 3 there was a significant (*α* = 0.05) correlation between the SPAD measurements under both Fe regimes (Figure 2). These observations are most likely related to the fact that leaf no. 3 was formed during the early vegetative growth phase when Fe was supplied in the preculture. This also allowed plants to build up an Fe reservoir in the root apoplast which can be an important Fe source during subsequent growth [34]. The detection of loci being important for an efficient depletion of the apoplastic Fe reservoir still need to be determined. Furthermore, the loci causing the differences between both Fe regimes in the relative chlorophyll contents of SP4 to SP6 may also merit further investigations respecting as they are likely to contribute to improved Fe efficiency and thus might be valuable in breeding programs. For this reason QTL mapping is the method of choice to identify those loci that allow improving Fe efficiency in maize.

### QTL and gene expression analyses

Besides the general analyses of the physiological and morphological traits evaluated under two Fe regimes for 85 IRILs we combined this phenotypic information with 1652 genetic markers for QTL mapping. Under Fe deficiency the traits SP3, RW, and H_2_O showed the highest number of QTLs (7 - 8) explaining between < 0.1% to 15% of the phenotypic variance (%r^2^) (Table 3). In contrast, SP4 to SP6 showed with 3 to 4 QTLs the highest %r^2^ of 34.0%. This observation suggests that under Fe deficiency SP4 to SP6 are of lower genetic complexity which increases the probability to identify in these QTL confidence intervals genes contributing largely to the natural variation in Fe homeostasis-related traits.

According to the genome sequence of the QTL intervals of SP3, SP5, and SP6 measured under Fe deficiency, these intervals include among others a Ferredoxin class gene (GRMZM2G043162) on chromosome 4 (Figure 3, Table 2). Ferredoxin transcript and protein levels strongly decrease under Fe deficiency [35] suggesting that ferredoxins respond sensitively to the Fe nutritional status. The sequence of the Ferredoxin class gene found in the above-mentioned QTL interval was not homologous to the maize ferredoxins FDX1, FDX2, FDX3, or FDX5 characterized by [36] or to FDX6. We assume that the detected Ferredoxin class gene is, like the other homologs, involved in the capture and distribution of reducing equivalents derived from photosynthetic electron transport chain in chloroplasts. However, more than the other homologs, this Ferredoxin class gene may be a candidate gene conferring differential chlorosis tolerance among maize lines. Targeted biochemical and metabolite analyses will be necessary to validate whether Ferredoxin class gene functions are crucial for chlorosis tolerance.

Under adequate Fe supply, we observed in the QTL intervals of SP3 to SP6 another putative ferredoxin gene that was located on chromosome 1 (Figure 3, Table 3). This ferredoxin gene is most likely an ortholog to *PETF* (GRMZM2G359127) in Chlamydomonas, which is upregulated under Fe sufficiency [37]. The precise function of this ferredoxin homolog in maize is still unclear. However, the corresponding maize protein sequence revealed a homology of 64% with the *PETF* gene of the cyanobacterium *Fischerella* (data not shown). As studies in algae have indicated that multiple ferredoxin isoforms allow for the allocation of reduction equivalents to specific metabolic pathways in the chloroplast, the putative maize ferredoxin PETF might be particularly relevant not only for electron transport in the presence or excess of Fe but also for intraspecific variation in this function.

Under Fe-deficient growth conditions, we detected in the QTL intervals for branching at the terminal 5 cm root (BTR), SP5, and SP6 the gene encoding the metal tolerance protein 8 (*MTP8*) (GRMZM2G116831) and in the QTL interval of NEC the *MTP4* (GRMZM2G118497) gene on chromosome 1 (Figure 3, Table 2). Schaaf et al., 2004 [19] showed that the maize phytosiderophore-transporter YS1 is able to transport besides Fe also other phytosiderophore-chelated metals across the membrane, which may contribute to the typical accumulation of metals in Fe-deficient maize plants [38,39]. Hanikenne et al., 2005 [40] and Talke et al., 2006 [41] showed that MTPs are necessary for the detoxification of excess metals by sequestering them from the cytoplasm to the vacuole. The detection of the MTPs in the QTL confidence intervals supported the notion that maize has to cope not only with Fe deficiency but also with excess accumulation of metals to prevent the cytoplasm from metal toxicity. In this regard, the results of our study suggested that these two MTPs apparently contribute to the genetic variability of Fe deficiency-induced chlorosis in maize leaves. Furthermore, the trait increasing alleles of QTL intervals including *MTP8* and *MTP4* were provided by B73 as well as Mo17, respectively. Therefore, the combination of trait increasing alleles from both parental inbreds at different loci may cause the transgressive segregation in progenies which can be used to breed maize inbreds with a higher tolerance against excess metal accumulation during Fe starvation (Figure 1; Fe-deficient).

The QTL confidence intervals of SP4, SP5, and SP6 monitored in Fe-sufficient plants included the nicotianamine synthase 3 (*NAS3*) (GRMZM2G478568) gene on chromosome 1 (Figure 3, Table 3). This observation is in accordance with results of [42] who showed that the NAS3 protein was exclusively present under Fe-sufficient growth conditions. The corresponding protein is important for NA-mediated Fe chelation under adequate or even excess Fe provision to prevent the formation of reactive oxygen species via the Fenton reaction and thus to maintain Fe homeostasis in the cytosol [21,43]. The gene expression analyses for *NAS3* showed a significant (*α* = 0.05) three times higher transcript level for B73 compared to Mo17 at adequate Fe growth conditions (Figure 4). Furthermore, the transcript level of *NAS3* in Mo17 did not vary significantly between adequate and deficient Fe supply. This, in turn, is in accordance with the observation that the trait-increasing allele in the QTL was provided by B73.

It was proposed by [11] that expression patterns of Fe homeostasis-related genes in grasses are regulated according to the Fe status and by sensing mechanisms mediated by the transcription factors IDEF1 and IDEF2. Furthermore, these authors showed that the expression pattern of *OsNAS3*, which is the ortholog of *ZmNAS3*[42], was downregulated during Fe limitation. Taken together, these observations suggest that the transcriptional regulation of the Mo17 allele of *NAS3* might carry a disadvantageous mutation, e.g. in the upstream regulators or *cis*-acting elements of *NAS3*.

In the QTL interval for the trait NEC under Fe-deficient conditions (chromosome 1) we detected the *DMAS1* (GRMZM2G060952) gene, which is essential for the last step of the phytosiderophore biosynthesis in maize [15]. Since the capacity for the synthesis and release of phytosiderophore in graminaceous plants is strongly determining chlorosis tolerance under Fe-limiting conditions [44], DMAS1 is likely to play a key role for efficient Fe acquisition irrespective of which Fe source is provided [45].

With regard to the DMAS1 gene expression, Mo17 showed under Fe deficiency a three times lower transcript level compared to B73 (Figure 4). Bashir et al., 2006 [15] showed that the *DMAS* genes of rice, barley, wheat, and maize were upregulated during Fe deficiency in roots allowing to enhance the production and secretion of PS. This indicates that Mo17 might have a disadvantageous allele in the promoter region of *DMAS1*. However, further genes which might be regulated by IDEF1 and IRO2 (*cf*. [46]) showed differences in expression levels between B73 and Mo17 under both Fe regimes, namely *NAS1* and the methionine cycle related genes *FDH1, IDI2, IDI4,* and *MTK* (Figure 4). Besides a weaker induction by an upstream regulator, this may also be due to a disequilibirium in Fe sensing leading to a more Fe-inefficient phenotype in Mo17.

The gene coding for the transcription factor *IDEF1* was not located in any QTL interval of our study and was not differentially expressed between B73 and Mo17 at either Fe regime (Figure 4). On the one hand, this might be explained by the fact that the resolution power for QTL mapping was too weak for the detection of a QTL including *IDEF1* in the confidence interval. However, the present results might also be explained by the presence of another upstream regulator in the *IDEF1*-dependent or an independent regulatory pathway. This unknown gene could be polymorphic between B73 and Mo17 and could be hidden within the confidence intervals of the corresponding QTL detected in our study in which no other obvious candidate gene was found. The detection of regulatory transcription factors essential for chlorosis-free growth under different Fe regimes would certainly benefit from additional analyses of quantitative trait loci with expression (eQTL) and protein (pQTL) data of genes modulating Fe homeostasis.

## Conclusions

With regard to Fe homeostasis, Mo17 contributed some advantageous alleles which caused in combination with the more advantageous genetic background of B73 a transgressive segregation in some IRILs. The morphological and physiological traits determined here indicated a moderate to high dependency on natural genetic variation suggesting a powerful basis for QTL mapping approaches. Based on our QTL mapping results, we were able to identify new putative candidate genes like Ferredoxin 1, putative ferredoxin *PETF*, *MTP4* and *MTP8* which have so far not been considered as relevant for efficient Fe homeostasis under both, low or high Fe concentrations. Furthermore, we characterized candidate gene expression and provided an insight into putative *trans*-acting regulation on candidate genes especially for *DMAS1, NAS3, NAS1, FDH1, IDI2, IDI4*, and *MTK*.

## Methods

### Plant material

The intermated recombinant inbred lines (IRILs) of the IBM population was used, which was derived from a cross of the maize parental inbreds B73 and Mo17 [47]. Due to the unavailability of seeds for the IRILs MO040, MO043, MO048, MO057, MO062, MO063, MO076, MO079, and MO344, a total of 85 IRILs were evaluated in our study.

### Culture conditions and evaluated traits

Maize seeds were sterilized in a 3% NaClO solution for 3 minutes and then treated with 60°C hot water for 5 minutes. Afterwards, seeds were placed between two filter paper sheets moistened with saturated CaSO_4_ solution for germination in the dark at room temperature. After 6 days, the germinated seeds were transplanted to a continuously aerated nutrient solution with nutrient concentrations as described by [48]. The plants were supplied with 100 *μ*M Fe(III)-EDTA for 7 days. From day 14 to 28, plants were supplied with 10 (Fe-deficient) or 300 (Fe-sufficient) *μ*M Fe(III)-EDTA. The nutrient solution was exchanged every third day. Plants were cultivated from day 7 to day 28 in a growth chamber at a relative humidity of 60%, a light intensity of 170 *μ*mol m^-2^ s^-1^ in the leaf canopy, and a day-night temperature regime of 16 h/24°C and 8 h/22°C, respectively.

Four plants of each genotype were grown in one 5 L pot. All pots were arranged in a split-plot design in the growth chamber, where the two parental genotypes were included as checks. The entire experiment was replicated *b* = 3 times.

The relative chlorophyll content of leaf 3, 4, 5, and 6 (SP3, SP4, SP5, and SP6) was measured with a SPAD meter (Minolta SPAD 502) at day 25 for each individual plant. Furthermore, stress symptoms like branching at the terminal 5 cm of the root (BTR) and leaf necrosis (NEC) were recorded as a visual score on a scale from 1 (high trait expression) to 9 (low trait expression). Furthermore, the lateral root formation (LAT) was recorded on a scale from 1 (low trait expression) to 9 (high trait expression) at day 26. Additionally, the root length (RL), root weight (RW), and shoot length (SL) were measured for all plants in one pot as one sample and root samples were frozen immediately in liquid nitrogen at harvest on day 28. After drying the shoot material at 70°C, shoot dry weight (SDW), water content (H_2_O), and the ratio between shoot dry weight and shoot length (SDW/SL) was calculated.

A total of 21 IRILs were selected such that they represented the largest possible variation of trait values for SP5. For each of these IRILs, shoot samples of four plants were pooled so that each IRIL was represented by one sample for each of the three replicates. Afterwards, the samples were ground and Fe concentrations were measured using inductively coupled plasma optical emission spectrometry (iCAP 6000 SERIES, Thermo Fisher) according to [49].

### Quantitative RT-PCR analysis

Total RNA was extracted from roots of two replications of the parental inbreds B73 and Mo17 that were collected from both Fe regimes using RNeasy Plant Mini Kit (QIAGEN, Germany). Total RNA was treated with DNase (Ambion DNA-*free*, Invitrogen). Afterwards, cDNA synthesis was performed (SuperScript VILO, Invitrogen) and primers for candidate genes (Additional file 1: Table S1, Additional file 2: Table S2) were used for quantitative RT-PCR according to the manufacturer’s instructions (DyNAmo ColorFlash SYBR Green qPCR Kit) using *ACTIN1* (NM_001155179.1) to normalize relative transcript abundances of candidate genes (Table 2).

### Statistical analyses

The data of each Fe treatment were analyzed using the following mixed model:

$$y_{\text{ik}}=\mu +g_{i}+r_{k}+e_{\text{ik}},$$

where *y*_*ik*_ is the mean of four plants of the *i*th genotype in one pot of the *k*th replication, *μ* the general mean, *g*_*i*_ the effect of the *i*th genotype, *r*_*k*_ the effect of the *k*th replication, and *e*_*ik*_ the residual error.

To estimate adjusted entry means (AEM) for all genotypes, *g*_*i*_ and *r*_*k*_ were considered as fixed. Furthermore, *g*_*i*_ was considered as random to estimate the genotypic variance ( $\sigma_{g}^{2}$) and the error variance ( $\sigma_{e}^{2}$). All mixed model calculations were performed with ASReml [50].

The broad sense heritability *H*^2^ for each Fe regime was calculated as:

$$H^{2}=\frac{\sigma_{g}^{2}}{\sigma_{g}^{2}+\frac{\sigma_{e}^{2}}{b}}.$$

The AEM of all genotypes for all traits and Fe regimes were tested with a Kolmogorov-Smirnov test [51] for their normal distribution. Partial correlation coefficients were assessed between all pairs of traits [52]. Network analyses of the partial correlations were prepared according to [53].

### Genetic map

The publicly available genotypic data ( http://www.maizegdb.org/map.php) for the IRILs were used in our study. The genetic map positions of these markers on the IBM2 map are available ( http://www.maizegdb.org/map.php) and were the basis of our analyses. 336 markers were excluded that showed a highly significant (P < 0.001) distorted segregation (*cf*. [54]). The remaining 1652 markers were used for the QTL analyses. Missing genotypic information in our marker set was imputed as described by [55].

### QTL analyses

Due to the high number of available markers, cofactors could not be selected using standard stepwise regression. Therefore, the following procedure was applied for each trait. One random marker was selected from each bin. Multiple stepwise regression was used to select cofactors from this set of markers based on the Bayesian information criterion (BIC) [56]. This procedure was repeated 1000 times. The average number of selected cofactors across the 1000 times repetition was used as estimator of the number of bins to study in more detail. Out of these bins 100 markers were chosen randomly and the final set of cofactors based on BIC was selected.

For the QTL mapping adjusted entry means of the 3 experimental replications for each trait, Fe regime, and each of 85 IRILs were used. The QTL analysis was carried out using the multiple QTL mapping (MQM) procedure [57] implemented in the R package ‘qtl’ version 1.21-2 [58]. The QTL detection was performed with a 2 cM (centi Morgan) step size (*cf*. [59]).

A total of 1000 permutation runs were performed for each trait and Fe regime to determine the *α* = 0.05 experiment-wise type I error for a QTL [60]. The 95% Bayesian confidence interval was calculated for each QTL location [61]. The confidence interval was expanded to the nearest flanking markers and their physical map localization was derived from B73 RefGen_v2_sequence to be able to extract all putative genes from a defined interval.

If not stated differently, all analyses were performed using the statistical software R [62].

## Competing interests

The authors declare that they have no competing interests.

## Authors’ contributions

AB, CU, and JM carried out the hydroponic growth of maize genotypes, tissue collection, and phenotype evaluation. AB and JM performed the quantitative real time PCRs. RS collected the iron concentration data. AB analyzed the data. AB, BS, and NvW drafted the manuscript. All authors read and approved the manuscript.

## Supplementary Material

Additional file 1 **Table S1 Genes involved in Fe homeostasis, which were projected on the IBM2 genetic map of maize (Figure **4**) and for quantitative RT-PCR analysis in root tissue.** Click here for file

Additional file 2 Table S2 Primer list (forward: F, reverse: R) for qRT-PCR. Click here for file
